# Coronary Venous Lead Extraction

**DOI:** 10.19102/icrm.2017.080604

**Published:** 2017-06-15

**Authors:** Edmond M. Cronin

**Affiliations:** ^1^Hartford HealthCare Heart and Vascular Institute at Hartford Hospital, Hartford, CT; ^2^University of Connecticut School of Medicine, Farmington, CT

**Keywords:** Cardiac resynchronization therapy, coronary sinus, electrodes, implanted, lead extraction

## Abstract

The increasing number of cardiac resynchronization therapy devices implanted, coupled with the increasing incidence of cardiac implantable electronic device infection, has led to a greater need for extraction of coronary venous pacing leads. The objectives of this study were to review the indications, techniques and published results of coronary venous lead extraction. In this study, we searched PubMed using the search terms “lead extraction,” “coronary sinus,” “coronary venous,” “pacing,” and “cardiac resynchronization therapy” for relevant papers. The reference lists of relevant articles were also searched, and personal experience was drawn upon. Published success rates and complications were found to be similar to those reported for non-coronary venous leads in experienced centers. However, reimplantation success differs and can be limited by vessel occlusion postextraction. The available active fixation coronary sinus lead (Attain Starfix™; Medtronic, MN, USA) is a particularly complex lead to extract, whereas limited data on the newer active fixation leads (Attain Stability™, Medtronic, MN, USA) suggest that they are less challenging to remove. The study concluded that coronary venous lead extraction presents unique challenges, especially reimplantation, that require special consideration and planning to overcome.

## Introduction

Expanded indications for cardiac resynchronization therapy (CRT), both with and without an implantable cardioverter-defibrillator (ICD), have led to increasing numbers of implants.^1,2^ This, coupled with an increasing incidence of cardiac implantable electronic device (CIED) infection, has led to a more frequent need of transvenous lead extraction (TLE) of coronary venous leads. The coronary venous system includes the coronary sinus (CS) and its branches and has a highly variable anatomy,^3^ leading to lead courses that can vary considerably. This is in contrast to relatively uniform right atrial and right ventricular lead courses. In addition, patients with coronary venous leads are generally regarded as being sicker and more medically complex than those with single- or dual-chamber ICDs or pacemakers. Lead extraction in these patients therefore involves both careful periprocedural patient management and a thorough knowledge of anatomy and techniques.

## Methods

We searched PubMed using the search terms “lead extraction” OR “coronary sinus” OR “coronary venous” OR “pacing” OR “cardiac resynchronization therapy” for papers relevant to the topic published from the database’s inception until December 31, 2016. The reference lists of the retrieved papers were searched for additional citations, and personal and peer experience and communications from subject matter experts were also relied upon.

To report the total published experience of coronary venous lead extraction, papers reporting at least five coronary venous lead extractions were selected. Patient, procedural, and outcome variables were extracted. Standard definitions according to the 2009 Heart Rhythm Society Expert Consensus were applied where possible.^4^ Overlapping reports were excluded, with the largest series from each center included. Complications due to CS lead extraction were reported as far as it was possible to ascertain.

### Patient selection

Indications for lead extraction in general are outlined in the Heart Rhythm Society Consensus Statement,^4^ and indications for coronary venous lead extraction mirror these. Specific indications for coronary venous lead extraction that are rarely seen with right atrial or right ventricular leads include phrenic nerve stimulation. Functional leads should not be the first choice for extraction for access, given the difficulty of reimplantation that is often encountered, as discussed below.

Although indications for extraction mirror those for patients with single- and dual-chamber devices, the patient population with CRT devices generally presents with more advanced cardiac dysfunction and cardiac and non-cardiac comorbidities, and particular consideration should be given to this in selecting patients for extraction. That said, indications for extraction likely occur more frequently in CRT patients, given the higher incidence of CIED infection,^5^ lead failure (given the higher number of leads), and venous occlusion or stenosis.^6–8^

### Preprocedural considerations

Minimum training and facility recommendations are outlined in the Heart Rhythm Society Consensus Statement,^4^ and apply to coronary venous lead extraction as well as other leads. However, additional consideration should be given to the fact that patients with CRT devices are often more complex than those with simpler systems; therefore, extraction should be undertaken in centers with specific expertise and capabilities. Additional resources may include the availability of cardiothoracic surgery with experience in the placement of an epicardial left ventricular lead in the event of failure to reimplant with a transvenous left ventricular lead, and mechanical cardiac support such as extracorporeal membrane oxygenation (ECMO) and percutaneous left ventricular support devices in the event of periprocedural hemodynamic deterioration.

### General approach

In cases such as infection, in which all leads are to be removed, the sequence of leads to be extracted is determined by various factors, usually starting with the most recently placed lead, or whichever is anticipated to be the easiest to remove. Given the small diameter and lack of active fixation mechanism in most coronary venous leads, these may be more likely to be extracted with traction alone as compared with, for instance, ICD leads. Transvenous extraction techniques for coronary venous leads and leads in general have recently been reviewed.^9,10^ Typically, controlled traction is first applied, with a standard stylet in the case of recently implanted leads, and with a locking stylet in the case of older leads. Care should be taken not to extend the stylet beyond the tip of the CS lead, which in contrast to virtually all other leads, is designed to be delivered over a guidewire and therefore has an open lumen distally. Branch perforation can occur if a stylet is advanced through this. If traction is insufficient, mechanical or powered sheaths can be used over a locking stylet with a suitably prepared lead.^9,10^ Currently, the most commonly used powered sheath in the United States is the excimer laser sheath (GlideLight™ and SLS™ II, Spectranetics, Colorado Springs, CO, USA). The 12-Fr sheath is suitable for currently available coronary venous sheaths, but larger sheaths may be required when working with early models or may be used for other leads within the same procedure. There are limited published data on the Evolution^®^ mechanical rotating tip sheath (Cook Medical, Bloomington, IN, USA) for coronary venous lead extraction^11–14^ and a single series on the TightRail™ sheath (Spectranetics, Colorado Springs, CO, USA).^15^ Nonetheless, these tools are useful options, either alone or in selected cases, or as adjunctive tools for use in areas of lead binding or heavy calcification. Polypropylene mechanical dilator sets may be used, such as those made by Cook Vascular Inc. (Leechburg, PA).^16^ Finally, CS delivery systems used to implant coronary venous leads, such as the Attain™ range (Medtronic, Minneapolis, MN), either in original form or modified by cutting off the distal soft tip, have been used to remove leads.^17^ These have the advantage of being specifically shaped for CS access.

### Alternative approaches

Coronary venous leads can be extracted from the femoral route as described by Bongiorni et al.^16^ This involves freeing up the lead from the implant vein (usually the left subclavian system), snaring the lead from the right femoral vein, and then proceeding with extraction with either manual traction or mechanical sheaths. The advantage of this route is that traction may be transmitted more directly to the tip of the lead, as the traction direction in this case better aligns with the course of the lead than from the superior approach, in which the direction of traction applied may be almost 180 degrees from that in which the lead courses in the CS. One disadvantage is the necessity of operating from two sites; however, this can be an adjunctive approach if the superior vein approach fails. As fibrous binding sites occur most commonly in the superior veins, some degree of dissection from the implant vein will almost always be necessary.^16^

### Outcomes of coronary venous lead extraction

Despite the greater anatomic complexity of coronary venous leads, extraction success rates in published series are comparable to general lead extraction experiences. This suggests that this complexity is balanced by the thinner diameter, lack of active fixation mechanism (in most leads), and the lack of defibrillator coils (especially superior vena cava coils) of coronary venous leads. A summary of published series of more than five coronary venous lead cases is presented in **Table 1.**^14–16,18–30^ The outcomes of active fixation lead extraction are also described separately below.

### Complications of coronary venous lead extraction

Complications include most commonly those generic to right atrial or ventricular leads, including vascular injury of the great vessels, causing hemothorax, or myocardial injury, leading to tamponade; pneumothorax, hematoma, and anesthetic complications; and lead dislodgement if reimplantation is performed. Complications specific to coronary venous lead extraction include avulsion or tearing of the CS or its branches, which has two potential consequences. First, bleeding from the coronary sinus could cause tamponade and could be difficult to localize and treat with an open surgical approach, given its posterior location. However, this specific mechanism of tamponade has, to this author’s knowledge, only been reported in the extraction of active fixation coronary venous leads.^28^ This may be due to the relative infrequency of fibrous binding sites within the CS,^16^ the small diameter of coronary venous leads (resulting in a smaller amount of fibrosis), and the small or even negative pressure gradient between the coronary venous system and the pericardial space. The second consequence is the impact on the ability to reimplant a coronary venous lead.

### Reimplantation postextraction

Many patients will undergo reimplantation of a CRT system postextraction, either during the same procedure or in a later procedure if infection is present. This may be limited by occlusion of the branch vessel or even the CS itself caused by avulsion or dissection when the fibrous tissue adherent to the lead is disturbed. In a study of 10 patients, CS venograms performed following lead extraction revealed that the original implant vein was usable in only 50% of cases. Furthermore, reimplantaiton was unsuccessful in 29% due to occlusion of the branches or CS.^21^ Another series of 90 patients reported successful reimplantation in 86 (95.6%), with occlusion of the branch in two, occlusion of the CS in one, and high threshold and phrenic nerve stimulation in one.^31^ Lastly, in a series of 173 patients whose CRT systems were extracted due to infection, contralateral reimplantation was unsuccessful in 19 of 107 (17.8%), usually due to the absence of suitable branches.^32^

### Active fixation coronary venous lead extraction

**Figure 1:**
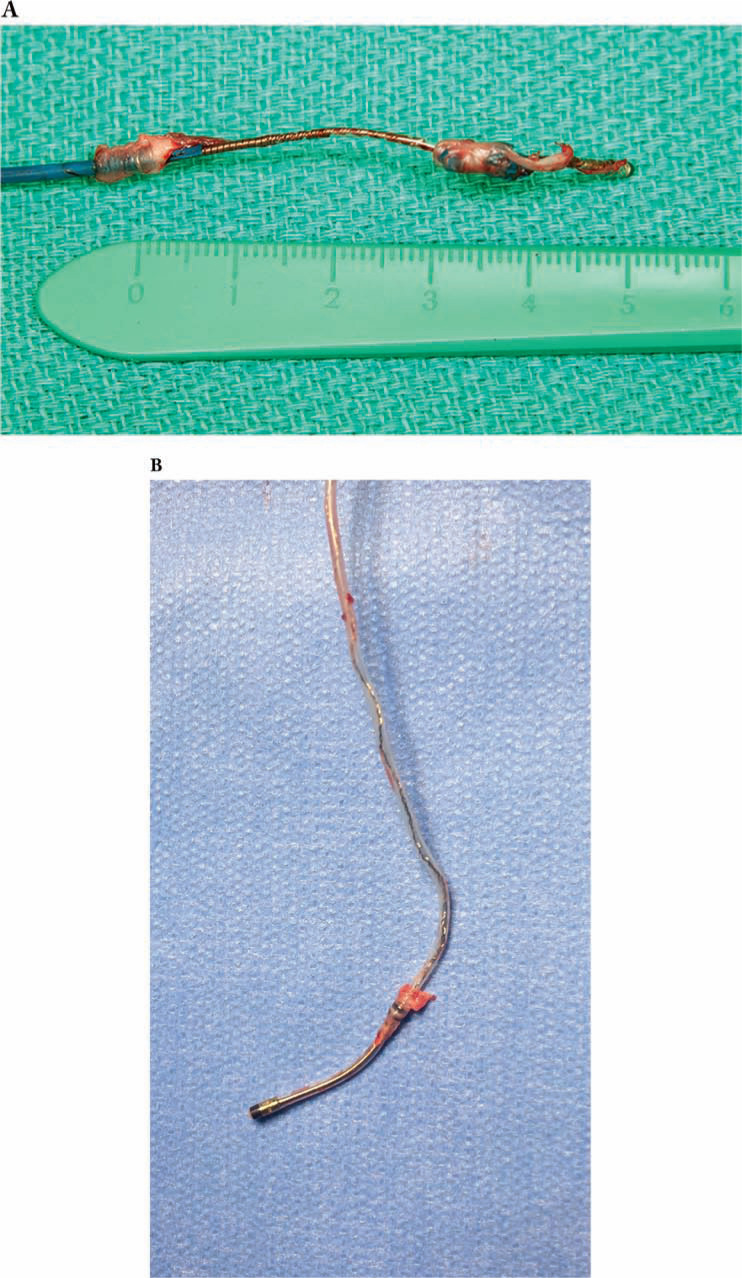
**A:** Fibrous ingrowth around an Attain Starfix™ 4195 (Medtronic, Minneapolis, MN, USA) lead with an implant duration of just over one year. **B:** A typical example of fibrous ingrowth around the proximal electrode of an Attain Ability™ 4196 (Medtronic, Minneapolis, MN, USA) lead with an implant duration of almost four times as long. Note the marked difference in the degree of fibrosis.

There are essentially two active fixation coronary venous leads with markedly different extraction considerations and handling difficulties due to their unique design features. The Attain Starfix™ model 4195 (Medtronic, Minneapolis, MN, USA) is a 5-Fr unipolar lead with extendable plastic fixation lobes that are deployed in the coronary venous branch to stabilize the lead. This leads to considerable difficulty during extraction, as vigorous fibrous ingrowth involving the extended lobes has been universally described.^33^ This has led to failure of retraction of the lobes, even shortly after implantation.^35^ In the investigational device exemption (IDE) trial, which led to approval in the United States, extraction (as defined by the Heart Rhythm Society’s Consensus Statement^4^) was attempted in seven cases at a mean of 734 days (range, 478 to 941 days), with partial or complete lobe retraction achieved in four of six cases reported, repositioning achieved in one lead, and extraction completed in two, with the remaining four abandoned.^34^ However, extraction tools were used in only three cases. In a prospective manufacturer-sponsored study comparing the extraction of the Attain Starfix™ (Medtronic, Minneapolis, MN, USA) leads to other coronary venous leads, the increased complexity of removal of the Attain Starfix™ (Medtronic, Minneapolis, MN, USA) lead was clear. Despite the Attain Starfix™ (Medtronic, Minneapolis, MN, USA) likely having a shorter implant duration (not reported, but leads in place less than six months were included), success was less (93% vs. 98.8%), major complications were twice as common, and one death occurred.^35^ Several case series and reports have also described significant challenges due to fibrous ingrowth around the active fixation lobes **(Figures 1A and 1B)**,^33,36,37^ with near-universal requirements for powered sheaths to be used for successful extraction, and a frequent necessity to advance the extraction sheath into the CS, which is much less common in cases of passive fixation leads. A small single-center comparison also found a lower success rate compared with passive fixation leads (50% vs. 100%), despite significantly shorter implant duration.^31^

**Figure 2:**
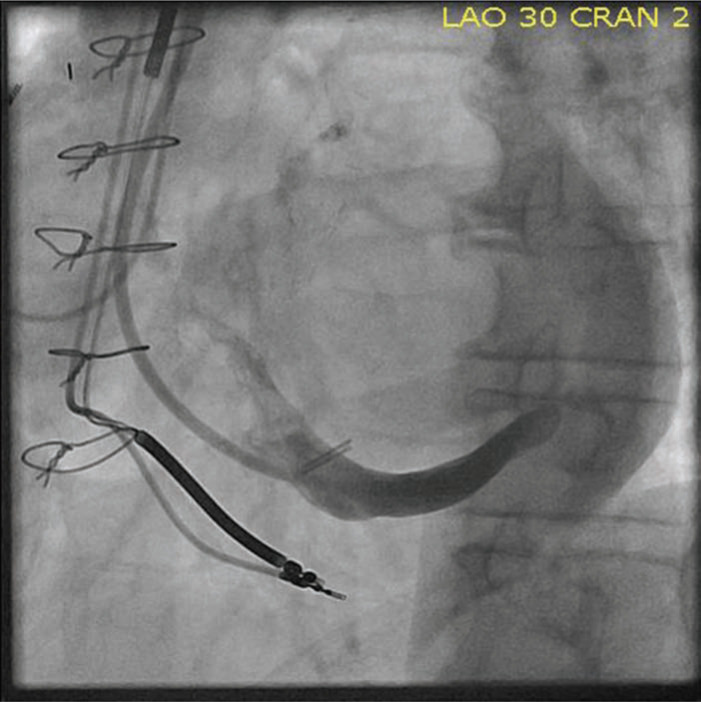
Occlusion of the distal coronary sinus postextraction of an Attain Starfix™ 4195 lead (Medtronic, Minneapolis, MN, USA). Limitation of transvenous reimplantation is particularly relevant after extraction of this lead.

Lessons learned from Attain Starfix™ (Medtronic, Minneapolis, MN, USA) lead extraction include the usefulness of advancing the extraction sheath within the CS to provide local countertraction. The mechanical or laser mechanisms of power sheaths should only be activated within the CS with extreme caution due to the risk of perforation and subsequent tamponade.^29,35^ A further complication of Attain Starfix™ (Medtronic, Minneapolis, MN, USA) lead extraction is the effect it has on reimplantation options. In our series of four leads, in which reimplantation was attempted in each case, the original implant vein was occluded in three and the main CS in the fourth case **(Figure 2)**, and reimplantation was successful in only two (50%) cases.^33^ This is in comparison to a reimplantation success rate of 82.8% of our large series of mostly passive fixation coronary venous lead extraction.^26^ The unipolar Attain Starfix™ (Medtronic, Minneapolis, MN, USA) lead has been made largely obsolete with the emergence of quadripolar leads, which allow for more stable distal positioning within a branch, while still allowing for pacing from a basal location, and the newer model active fixation lead. Given the complexities described above, extraction of this lead should only be completed by operators with experience and thorough familiarity with its construction and extraction techniques.

The Attain Stability™ (Medtronic, Minneapolis, MN, USA) family of leads is the current generation of active fixation coronary venous leads. Instead of deployable plastic lobes near the tip, the fixation mechanism is a small helix on the body of the lead designed to engage into the vein wall with rotation of the lead, thereby fixing the lead with minimal trauma or fibrous ingrowth. The helix is designed to straighten out when traction is placed on it during extraction. The first model, 20066, was a 4-Fr bipolar over-the-wire lead based on the passive fixation Attain Ability™ model 4196 (Medtronic, Minneapolis, MN, USA). Several authors have reported extraction of this lead at up to eight months’ implant duration without difficulty, using a technique of counterclockwise turning followed by manual traction.^38–40^ These reports also confirmed that the helix elongates with traction, facilitating extraction.^38,39^ Only one case described reimplantation after seven months’ implant duration, in which the branch was subtotally occluded but was recanalized using an angioplasty balloon and a lead reimplanted in the same location.^39^ This would not be unusual after coronary venous lead implantation, and further data are needed to assess the implications for reimplantation of this novel lead. A quadripolar version of the lead is currently undergoing clinical trials in the United States.

### Future directions

Limited data have been reported on the extraction of the newer quadripolar coronary venous leads; however, in this author’s experience, they behave similarly to passive fixation bipolar and unipolar leads. One relevant design feature is that the Medtronic series are non-isodiametric, with electrodes larger than the lead body. This may be offset by the fact that each electrode is also steroid eluting, which may reduce the amount of fibrous reaction surrounding the electrode.

Future technological innovations likely to be relevant to coronary venous lead extraction include the development of leadless pacing. Systems including left ventricular pacing via the coronary venous system are already in development. The extraction of such electrodes is likely to be complex, perhaps taking the form of a snare delivered via a sheath, which would then be used for countertraction.

## Conclusions

Coronary venous lead extraction involves patient, lead, anatomical, and reimplant considerations, which render it more complex than the extraction of simpler pacing systems. This is somewhat offset by more frequent removal with simple traction. Active fixation leads and limited reimplantation options are specific considerations requiring careful planning.

## Figures and Tables

**Table 1:** Published Series of CS Lead Extraction (Involving At Least Five Leads)

Author	Number of Leads	Mean (Range) Implant Duration, Months	Complete Success	Clinical Success	Major Procedural Complications	Transvenous Reimplant Success (When Attempted)
Di Cori 2012^16†^	147	29	99%	99%	1 (0.7%)	N/R
Tyers 2003^18^*	13	67.8 (0.3-320.4)	100%	100%	0 (0%)	66.7%
Kasravi 2005^19^	14	17 (2-43)	100%	100%	0 (0%)	92.9%
De Martino 2005^20^	12	13.9 (3-46)	100%	100%	0 (0%)	100%
Burke 2005^21*^	10	10.5 (3-59)	100%	100%	0 (0%)	70%
Hamid 2009^22^	32	26.5 (1-116)	100%	100%	0 (0%)	100%
Williams 2011^23*†^	60	35.8 (1-116)	98%	98%	1 (1.4%)	100%
Sheldon 2012^24^	125	18.5 (0.26–98.9)	99%	99%	9 (7.2%)	92.2%
Chu 2012^25^	24	29.5 (3-78)	95.8%	100%	N/R	N/R
Lisy 2013^27^	41	17.2 (0-104.9)	100%	100%	0 (0%)	N/R
Starck 2013^28^	27	33.3 (1-76)	94.7%	100%	0 (0%)	100%
Kypta 20 1 5^29†^	et	46.5	100%	100%	1 (16.6%)	N/R
Pecha 2016^30^	22	9.9(1-30.1)	19	19	0	71.4%
Rickard 2012^32^	173	22.3	97.7%	100%	1.2%	82.2%
Crossley 20 1 6^35†^	215	N/R	N/R	95%	16 (7.4%)	N/R
Maytin 20 1 2^37†^	12	14.2 (2.3-23.6)	92%	92%	0 (0%)	N/R
Total	933		97.5%	98.1%	2.3%	88.7%

Overlapping reports have been omitted, and the largest or most completely reported series from each center is included. The complications reported are due to CS lead extraction, as far as it was possible to discern. The complications were defined upon review of the paper in accordance with the Heart Rhythm Society consensus statement, and therefore may differ slightly from those in individual reports. ^*^Series includes CS defibrillation coils. ^†^Series includes Attain Starfix™ 4195 (Medtronic, Minneapolis, MN, USA) extractions. A single Attain Starfix™ (Medtronic, Minneapolis, MN, USA) lead was included in both Di Cori et al. 2012^16^ and Maytin et al. 2012.^37^
